# 1-(4-Meth­oxy­phen­yl)pyrrolidine-2,5-dione

**DOI:** 10.1107/S1600536813022460

**Published:** 2013-08-17

**Authors:** Muhammad Sirajuddin, Saqib Ali

**Affiliations:** a Department of Chemistry, Quaid-i-Azam University, Islamabad, Pakistan

## Abstract

In the title compound, C_11_H_11_NO_3_, the di­hydro­furan-2,5-dione ring has a shallow envelope conformation, with one of the methyl­ene C atoms displaced by 0.216 (1) Å from the other atoms. These near-planar atoms subtend a dihedral angle of 55.88 (8)° with the benzene ring. In the crystal, C—H⋯O hydrogen bonds link the mol­ecules into [010] chains.

## Related literature

For related structures, see: Sirajuddin *et al.* (2012 ▶); Tahir *et al.* (2012 ▶).
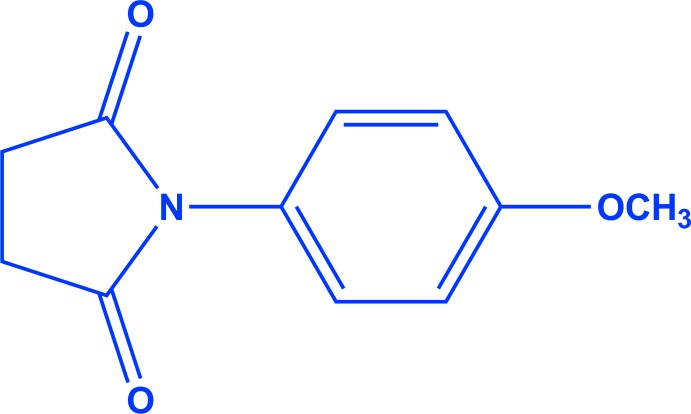


## Experimental

### 

#### Crystal data

C_11_H_11_NO_3_
*M* *_r_* = 205.21
Monoclinic, 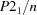

*a* = 9.3684 (7) Å
*b* = 6.6146 (4) Å
*c* = 16.0720 (11) Å
β = 99.939 (4)°
*V* = 981.01 (12) Å^3^
*Z* = 4
Mo *K*α radiation
μ = 0.10 mm^−1^
*T* = 296 K
0.32 × 0.25 × 0.22 mm

#### Data collection

Bruker Kappa APEXII CCD diffractometer
Absorption correction: multi-scan (*SADABS*; Bruker, 2005 ▶) *T*_min_ = 0.968, *T*_max_ = 0.978
7585 measured reflections
1927 independent reflections
1626 reflections with *I* > 2σ(*I*)
*R*_int_ = 0.018

#### Refinement

*R*[*F* ^2^ > 2σ(*F* ^2^)] = 0.034
*wR*(*F* ^2^) = 0.094
*S* = 1.04
1927 reflections
138 parameters
H-atom parameters constrained
Δρ_max_ = 0.14 e Å^−3^
Δρ_min_ = −0.14 e Å^−3^

### 

Data collection: *APEX2* (Bruker, 2005 ▶); cell refinement: *SAINT* (Bruker, 2005 ▶); data reduction: *SAINT*; program(s) used to solve structure: *SHELXS97* (Sheldrick, 2008 ▶); program(s) used to refine structure: *SHELXL2012* (Sheldrick, 2008 ▶); molecular graphics: *ORTEP-3 for Windows* (Farrugia, 2012 ▶) and *PLATON* (Spek, 2009 ▶); software used to prepare material for publication: *WinGX* (Farrugia, 2012 ▶) and *PLATON*.

## Supplementary Material

Crystal structure: contains datablock(s) I, global. DOI: 10.1107/S1600536813022460/hb7117sup1.cif

Structure factors: contains datablock(s) I. DOI: 10.1107/S1600536813022460/hb7117Isup2.hkl

Supplementary material file. DOI: 10.1107/S1600536813022460/hb7117Isup3.cml

Additional supplementary materials:  crystallographic information; 3D view; checkCIF report

## Figures and Tables

**Table 1:** Hydrogen-bond geometry (Å, °)

*D*—H⋯*A*	*D*—H	H⋯*A*	*D*⋯*A*	*D*—H⋯*A*
C2—H2⋯O3^i^	0.93	2.50	3.1666 (17)	129
C5—H5⋯O2^ii^	0.93	2.47	3.3245 (17)	152

Symmetry codes: (i) 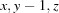; (ii) 
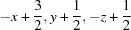
.

**Fig. 1.**
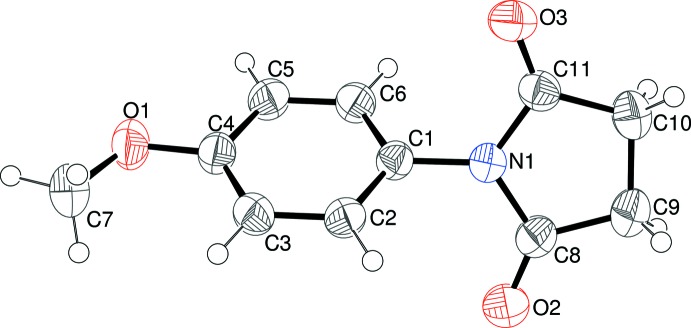
View of the title compound with displacement ellipsoids drawn at the 50% probability level.

**Fig. 2.**
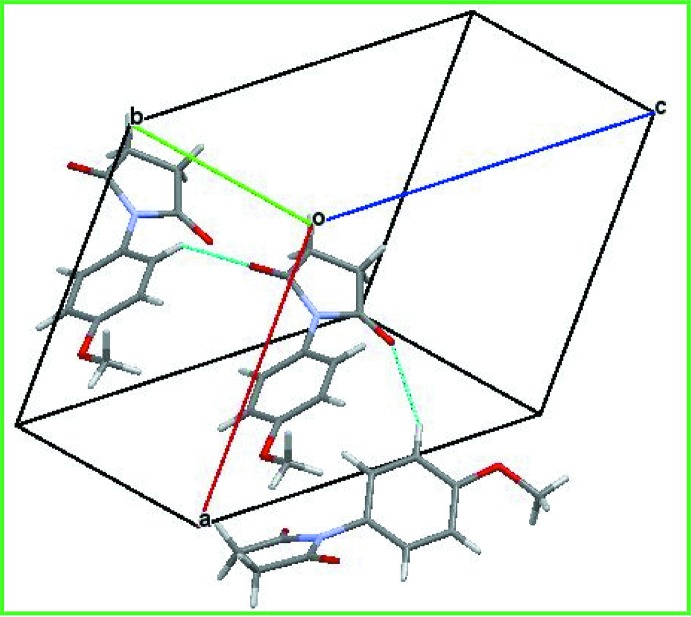
The partial packing, which shows that molecules form dimers due to C—H···O bondings.

## supplementary crystallographic information

### 1. Experimental

Equimolar quantities of 4-methoxyaniline and dihydrofuran-2,5-dione were stirred and refluxed in acetic acid for 4 h. The solution was kept at room temperature for 24 h which afforded colourless prisms of the title compound.

### 2. Refinement

The H atoms were positioned geometrically (C—H = 0.93–0.96 Å) and refined as riding with *U*_iso_(H) = *xU*_eq_(C), where *x* = 1.5 for methyl and *x* = 1.2 for other H atoms.

### Crystal data

**Table:**

C_11_H_11_NO_3_	*Z* = 4
*M**_r_* = 205.21	*F*(000) = 432
Monoclinic, *P*2_1_/*n*	*D*_x_ = 1.389 Mg m^−^^3^
*a* = 9.3684 (7) Å	Mo *K*α radiation, λ = 0.71073 Å
*b* = 6.6146 (4) Å	µ = 0.10 mm^−^^1^
*c* = 16.0720 (11) Å	*T* = 296 K
β = 99.939 (4)°	Prism, colourless
*V* = 981.01 (12) Å^3^	0.32 × 0.25 × 0.22 mm

### Data collection

**Table:**

Bruker Kappa APEXII CCD diffractometer	1927 independent reflections
Radiation source: fine-focus sealed tube	1626 reflections with *I* > 2σ(*I*)
Detector resolution: 8 pixels mm^-1^	*R*_int_ = 0.018
ω scans	θ_max_ = 26.0°, θ_min_ = 2.4°
Absorption correction: multi-scan (*SADABS*; Bruker, 2005)	*h* = −11→11
*T*_min_ = 0.968, *T*_max_ = 0.978	*k* = −8→7
7585 measured reflections	*l* = −19→19

### Refinement

**Table:**

Refinement on *F*^2^	Hydrogen site location: inferred from neighbouring sites
Least-squares matrix: full	H-atom parameters constrained
*R*[*F*^2^ > 2σ(*F*^2^)] = 0.034	*w* = 1/[σ^2^(*F*_o_^2^) + (0.0409*P*)^2^ + 0.2498*P*] where *P* = (*F*_o_^2^ + 2*F*_c_^2^)/3
*wR*(*F*^2^) = 0.094	(Δ/σ)_max_ < 0.001
*S* = 1.04	Δρ_max_ = 0.14 e Å^−^^3^
1927 reflections	Δρ_min_ = −0.14 e Å^−^^3^
138 parameters	Extinction correction: *SHELXL2012* (Sheldrick, 2012), Fc^*^=kFc[1+0.001xFc^2^λ^3^/sin(2θ)]^-1/4^
0 restraints	Extinction coefficient: 0.047 (4)

### Special details

**Table:**

Geometry. All e.s.d.'s (except the e.s.d. in the dihedral angle between two l.s. planes) are estimated using the full covariance matrix. The cell e.s.d.'s are taken into account individually in the estimation of e.s.d.'s in distances, angles and torsion angles; correlations between e.s.d.'s in cell parameters are only used when they are defined by crystal symmetry. An approximate (isotropic) treatment of cell e.s.d.'s is used for estimating e.s.d.'s involving l.s. planes.

### Fractional atomic coordinates and isotropic or equivalent isotropic displacement parameters (Å^2^)

**Table:**

	*x*	*y*	*z*	*U*_iso_*/*U*_eq_	
O1	0.73798 (12)	−0.12759 (16)	0.03467 (7)	0.0594 (3)	
O2	0.52321 (14)	−0.03671 (17)	0.38782 (7)	0.0685 (4)	
O3	0.38050 (12)	0.48906 (15)	0.21179 (6)	0.0588 (3)	
N1	0.48031 (11)	0.21443 (15)	0.28804 (6)	0.0397 (3)	
C1	0.54572 (13)	0.12100 (19)	0.22333 (7)	0.0378 (3)	
C2	0.50543 (14)	−0.0707 (2)	0.19526 (8)	0.0418 (3)	
H2	0.4367	−0.1412	0.2192	0.050*	
C3	0.56658 (14)	−0.1592 (2)	0.13165 (8)	0.0441 (3)	
H3	0.5385	−0.2882	0.1125	0.053*	
C4	0.66963 (14)	−0.0546 (2)	0.09689 (8)	0.0427 (3)	
C5	0.71239 (14)	0.1368 (2)	0.12663 (9)	0.0461 (3)	
H5	0.7830	0.2061	0.1039	0.055*	
C6	0.65080 (14)	0.2246 (2)	0.18962 (8)	0.0427 (3)	
H6	0.6797	0.3529	0.2094	0.051*	
C7	0.6833 (2)	−0.3084 (2)	−0.00666 (10)	0.0639 (4)	
H7A	0.6941	−0.4177	0.0333	0.096*	
H7B	0.7362	−0.3386	−0.0512	0.096*	
H7C	0.5825	−0.2911	−0.0299	0.096*	
C8	0.47536 (15)	0.1275 (2)	0.36638 (8)	0.0471 (3)	
C9	0.39873 (18)	0.2721 (2)	0.41557 (9)	0.0556 (4)	
H9A	0.4574	0.3009	0.4701	0.067*	
H9B	0.3067	0.2164	0.4244	0.067*	
C10	0.37563 (16)	0.4609 (2)	0.36233 (9)	0.0494 (4)	
H10A	0.2764	0.5078	0.3572	0.059*	
H10B	0.4402	0.5679	0.3869	0.059*	
C11	0.40880 (14)	0.39934 (19)	0.27787 (8)	0.0418 (3)	

### Atomic displacement parameters (Å^2^)

**Table:**

	*U*^11^	*U*^22^	*U*^33^	*U*^12^	*U*^13^	*U*^23^
O1	0.0720 (7)	0.0563 (6)	0.0574 (6)	−0.0022 (5)	0.0327 (5)	−0.0108 (5)
O2	0.0974 (9)	0.0604 (7)	0.0501 (6)	0.0251 (6)	0.0192 (6)	0.0146 (5)
O3	0.0802 (7)	0.0454 (6)	0.0510 (6)	0.0083 (5)	0.0116 (5)	0.0074 (5)
N1	0.0446 (6)	0.0388 (6)	0.0366 (6)	0.0017 (4)	0.0099 (4)	−0.0006 (4)
C1	0.0399 (6)	0.0397 (6)	0.0337 (6)	0.0018 (5)	0.0065 (5)	−0.0006 (5)
C2	0.0427 (7)	0.0406 (7)	0.0440 (7)	−0.0038 (5)	0.0125 (5)	0.0014 (5)
C3	0.0488 (7)	0.0380 (7)	0.0458 (7)	−0.0034 (6)	0.0090 (6)	−0.0048 (6)
C4	0.0447 (7)	0.0458 (7)	0.0388 (7)	0.0036 (6)	0.0110 (5)	−0.0010 (6)
C5	0.0450 (7)	0.0468 (8)	0.0494 (8)	−0.0058 (6)	0.0163 (6)	0.0017 (6)
C6	0.0453 (7)	0.0392 (7)	0.0436 (7)	−0.0054 (5)	0.0079 (5)	−0.0033 (5)
C7	0.0837 (11)	0.0598 (10)	0.0514 (9)	0.0052 (8)	0.0207 (8)	−0.0129 (7)
C8	0.0540 (8)	0.0498 (8)	0.0377 (7)	0.0036 (6)	0.0088 (6)	0.0021 (6)
C9	0.0657 (9)	0.0614 (9)	0.0430 (8)	0.0048 (7)	0.0183 (7)	−0.0035 (7)
C10	0.0496 (8)	0.0492 (8)	0.0502 (8)	0.0003 (6)	0.0110 (6)	−0.0116 (6)
C11	0.0440 (7)	0.0363 (6)	0.0447 (7)	−0.0034 (5)	0.0066 (5)	−0.0023 (6)

### Geometric parameters (Å, º)

**Table:**

O1—C4	1.3654 (16)	C5—C6	1.3773 (18)
O1—C7	1.4203 (18)	C5—H5	0.9300
O2—C8	1.2028 (17)	C6—H6	0.9300
O3—C11	1.2055 (16)	C7—H7A	0.9600
N1—C11	1.3905 (16)	C7—H7B	0.9600
N1—C8	1.3920 (17)	C7—H7C	0.9600
N1—C1	1.4350 (15)	C8—C9	1.5002 (19)
C1—C2	1.3766 (18)	C9—C10	1.508 (2)
C1—C6	1.3844 (18)	C9—H9A	0.9700
C2—C3	1.3850 (18)	C9—H9B	0.9700
C2—H2	0.9300	C10—C11	1.5003 (18)
C3—C4	1.3819 (18)	C10—H10A	0.9700
C3—H3	0.9300	C10—H10B	0.9700
C4—C5	1.3873 (19)		
			
C4—O1—C7	117.61 (11)	O1—C7—H7B	109.5
C11—N1—C8	112.27 (11)	H7A—C7—H7B	109.5
C11—N1—C1	123.43 (10)	O1—C7—H7C	109.5
C8—N1—C1	124.25 (11)	H7A—C7—H7C	109.5
C2—C1—C6	120.06 (11)	H7B—C7—H7C	109.5
C2—C1—N1	120.46 (11)	O2—C8—N1	124.27 (13)
C6—C1—N1	119.47 (11)	O2—C8—C9	127.83 (13)
C1—C2—C3	120.42 (12)	N1—C8—C9	107.89 (12)
C1—C2—H2	119.8	C8—C9—C10	105.29 (11)
C3—C2—H2	119.8	C8—C9—H9A	110.7
C4—C3—C2	119.58 (12)	C10—C9—H9A	110.7
C4—C3—H3	120.2	C8—C9—H9B	110.7
C2—C3—H3	120.2	C10—C9—H9B	110.7
O1—C4—C3	124.58 (12)	H9A—C9—H9B	108.8
O1—C4—C5	115.53 (12)	C11—C10—C9	104.87 (11)
C3—C4—C5	119.87 (12)	C11—C10—H10A	110.8
C6—C5—C4	120.36 (12)	C9—C10—H10A	110.8
C6—C5—H5	119.8	C11—C10—H10B	110.8
C4—C5—H5	119.8	C9—C10—H10B	110.8
C5—C6—C1	119.69 (12)	H10A—C10—H10B	108.8
C5—C6—H6	120.2	O3—C11—N1	124.39 (12)
C1—C6—H6	120.2	O3—C11—C10	127.96 (13)
O1—C7—H7A	109.5	N1—C11—C10	107.65 (11)
			
C11—N1—C1—C2	−123.12 (13)	N1—C1—C6—C5	−179.05 (11)
C8—N1—C1—C2	53.95 (17)	C11—N1—C8—O2	176.06 (14)
C11—N1—C1—C6	57.37 (16)	C1—N1—C8—O2	−1.3 (2)
C8—N1—C1—C6	−125.55 (14)	C11—N1—C8—C9	−2.76 (16)
C6—C1—C2—C3	−1.76 (19)	C1—N1—C8—C9	179.88 (12)
N1—C1—C2—C3	178.74 (11)	O2—C8—C9—C10	174.72 (15)
C1—C2—C3—C4	0.6 (2)	N1—C8—C9—C10	−6.52 (16)
C7—O1—C4—C3	10.8 (2)	C8—C9—C10—C11	12.49 (15)
C7—O1—C4—C5	−170.72 (13)	C8—N1—C11—O3	−168.93 (13)
C2—C3—C4—O1	179.31 (12)	C1—N1—C11—O3	8.5 (2)
C2—C3—C4—C5	0.8 (2)	C8—N1—C11—C10	11.02 (15)
O1—C4—C5—C6	−179.76 (12)	C1—N1—C11—C10	−171.59 (11)
C3—C4—C5—C6	−1.2 (2)	C9—C10—C11—O3	165.50 (14)
C4—C5—C6—C1	0.0 (2)	C9—C10—C11—N1	−14.44 (14)
C2—C1—C6—C5	1.44 (19)		

### Hydrogen-bond geometry (Å, º)

**Table:**

*D*—H···*A*	*D*—H	H···*A*	*D*···*A*	*D*—H···*A*
C2—H2···O3^i^	0.93	2.50	3.1666 (17)	129
C5—H5···O2^ii^	0.93	2.47	3.3245 (17)	152

Symmetry codes: (i) *x*, *y*−1, *z*; (ii) −*x*+3/2, *y*+1/2, −*z*+1/2.
